# Laminin functionalization of zirconia abutments modulates early oral biofilm formation in vitro

**DOI:** 10.1080/20002297.2026.2731523

**Published:** 2026-09-15

**Authors:** Romualdus Nugraha Catur Utomo, Alena L. Palkowitz, Philipp Schräder, Lin Gan, Hendrik Ballerstedt, Martin Zimmermann, Lars M. Blank, Horst Fischer, Stefan Wolfart, Taşkın Tuna

**Affiliations:** a Department of Prosthodontics and Biomaterials, RWTH Aachen University Hospital, Aachen, Germany; b Department of Dental Materials and Biomaterials Research, RWTH Aachen University Hospital, Aachen, Germany; c Genomic Facility, IZKF RWTH Aachen University Hospital - Aachen, Aachen, Germany; d Institute of Applied Microbiology (iAMB), Aachen Biology and Biotechnology (ABBt), RWTH Aachen University, Aachen, Germany

**Keywords:** Oral biofilm formation, dental abutment materials, extracellular matrix proteins, laminin, fibronectin, biofilm modulation, 16S rRNA gene analysis, cell viability, soft-tissue integration

## Abstract

**Background:**

Surface properties of dental implant abutment materials critically influence supragingival biofilm formation by determining the outcome of the early ‘race for the surface’, in which gingival epithelial cells and fibroblasts compete with bacteria for initial adhesion. This study hypothesized that biofunctionalization with extracellular matrix (ECM) proteins fibronectin (FN) or laminin (LN) modulates early oral biofilm formation on titanium alloy (Ti6Al4V) and yttria-stabilized zirconia (Y-TZP) abutments.

**Methods:**

Saliva-derived microcosm biofilms were grown on control and biofunctionalized discs. Biofilm biomass, viability, structure, microbial community composition and metabolic activity were assessed using microscopy-based methods, impedance flow cytometry, 16S rRNA sequencing and short-chain fatty acid analysis.

**Results:**

Laminin-functionalized Y-TZP showed the most pronounced delay in early biofilm formation. Biofilm accumulation was delayed during the first three days and was associated with the lowest number of viable cells on day 4, minimal extracellular polymeric substance formation, reduced *Veillonella* abundance (~9% on day 1) and decreased short-chain fatty acid production.

**Conclusion:**

ECM-based coatings do not exert bactericidal effects but transiently reshape early oral biofilm formation. Laminin-functionalized zirconia may act as a biologically selective interface that favors beneficial early host–microbe interactions, potentially creating a microbial environment more compatible with peri-implant soft-tissue health.

## Graphical abstract

**Figure:**
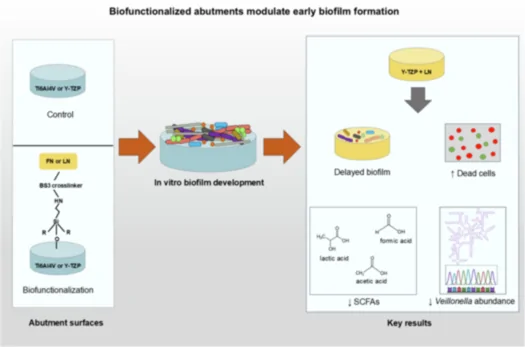

## Introduction

Dental implants have transformed modern dentistry by providing reliable restoration of function and esthetics through osseointegration—the direct structural and functional connection between bone and implant [1]. Despite high survival rates, implant failures still occur [2]. Primary failures due to insufficient osseointegration affect about 1–2% of patients, typically within the first months after placement [3], whereas secondary failures—often associated with peri-implantitis—are reported in approximately 5% of cases several years later [2,4], based largely on evidence from titanium implant systems. While osseointegration remains the biological foundation for implant stability and is generally well achieved with modern implant systems, the predominance of secondary, peri-implantitis-driven failures has increasingly shifted clinical attention toward the peri-implant soft tissue barrier surrounding the abutment, commonly referred to as soft tissue integration (STI) [5]. The integrity of this transmucosal barrier is now considered essential for protecting the underlying bone from microbial challenge and for maintaining long-term peri-implant health [5,6].

The peri-implant mucosa provides the first line of defense against microbial invasion but remains structurally and immunologically fragile. Lacking the periodontal ligament and cementum of natural teeth, it shows weaker connective-tissue anchorage, reduced vascularization and limited immune surveillance [7,8]. This vulnerability increases susceptibility to bacterial colonization and peri-implant inflammation, underscoring the need for implant–abutment surfaces that actively promote soft-tissue sealing while minimizing bacterial adhesion [6]. The choice of abutment material is therefore a key factor in shaping this early soft-tissue response. While titanium implants remain the clinical reference standard due to their well-documented long-term osseointegration and survival outcomes, zirconia-based implant systems are still being evaluated regarding their long-term clinical predictability, with a meta-analysis reporting a significantly (*p* = 0.02) increased risk (4.2 times (95% CI: 1.24; 14.17)) of early implant failure compared with titanium [9] —a pattern that contrasts with the failure profile described above, where late, peri-implantitis-associated complications predominate. Regardless of this osseointegration debate, zirconia materials—particularly in transmucosal and abutment components—have attracted increasing interest because of their biocompatibility and promising soft-tissue response, including lower plaque accumulation compared with titanium abutments without significant differences in inflammatory outcomes [10–12].

Given the focus of the present study on this transmucosal soft tissue–biofilm interface rather than endosseous integration, understanding the biological interplay among abutment surface, host tissue and early bacterial colonizers is of particular relevance.

In this context, material composition and surface design are key determinants of peri-implant soft tissue integration. Titanium alloys (Ti6Al4V) remain the clinical standard, whereas yttria-stabilized tetragonal zirconia (Y-TZP) has emerged as a promising metal-free alternative with favorable esthetic and soft-tissue-related properties [5]. Beyond bulk composition, surface chemistry, and topography strongly affect epithelial and fibroblast responses. Physical or chemical modifications, such as anodization [13–15], laser texturing [16–18] or plasma treatments [19–21], can improve wettability and cell attachment but provide limited biological signaling [5,22].

Bioinspired approaches therefore seek to combine antibacterial and regenerative properties by incorporating biologically active molecules. Strategies using peptides, growth factors or extracellular matrix (ECM) proteins—particularly fibronectin (FN) [23–25] and laminin (LN) [5,7] aim to promote integrin-mediated host-cell attachment and soft-tissue stability [26,27]. Among these, laminin-332 coatings have shown promise in reinforcing epithelial attachment via hemidesmosome formation, while fibronectin enhances fibroblast adhesion [28,29].

In our previous studies, we established a covalent biofunctionalization protocol for Ti6Al4V and Y-TZP using full-length ECM proteins, producing stable, hydrophilic coatings that supported gingival fibroblast adhesion and integrin expression [30]. The current study expands this research by exploring, for the first time, how these ECM coatings influence early bacterial colonization and metabolic activity within a saliva-derived oral biofilm model. We hypothesized that fibronectin- and laminin-functionalized abutment materials would reduce or delay bacterial adhesion, modulate biofilm maturation, and alter microbial metabolism compared with uncoated controls.

## Materials and methods

### Sample preparation of Ti6Al4V and Y-TZP

Cylindrical titanium alloy (Ti6Al4V) bars were procured from EWG E. Wagener (Germany). Ti6Al4V discs (9 mm in diameter and 1.5 mm in thickness) were fabricated by turning, followed by sequential grinding with silicon carbide abrasive papers of grit sizes No. 80, 800, 1,200 and 4,000 (ATM, Germany). Yttria-partially stabilized zirconia (Y-TZP) discs (TZ-3YSB-E powder, Tosoh, Japan) with diameter of approximately 12 mm and with thickness of 2.5–3 mm were manufactured by uniaxial dry pressing using a stainless-steel cylindrical mold. Subsequently, the Y-TZP discs were sintered at 1,500 °C for 2 h (Therm-Aix, Germany), followed by grinding with the diamond grinding discs ‘Galaxy Red’ and ‘Galaxy Blue’ and polishing with diamond pastes (ATM, Germany). To remove any organic residues, the ceramic discs were heated to 450 °C for 15 min. After the completed process, the Y-TZP discs exhibited a final diameter of 9 mm and a thickness of 1.5 mm, corresponding to an overall size reduction of approximately 25% in diameter relative to the pre-sintering dimensions, attributable to both sintering shrinkage and subsequent mechanical grinding and polishing. Finally, zirconia discs were disinfected by sonication in 70% ethanol (Schmittmann GmbH, Germany) and Milli-Q water (Sartorius, Germany).

Titanium discs were cleaned using a comparable protocol. Briefly, samples were ultrasonicated in 70% ethanol (Schmittmann GmbH, Germany) and Milli-Q water (Sartorius, Germany). No high-temperature treatment was applied due to different material properties. This procedure ensured a comparable level of surface cleanliness for subsequent experimental investigations.

Both specimen types were processed to achieve a comparable surface roughness of Ra < 0.2 µm, ensuring comparable surface conditions for biofilm formation across material groups. Surface topography was analyzed using a Keyence VK-X100 confocal laser scanning microscope (Keyence Deutschland, Neu-Isenburg, Germany).

### Pretreatment and silanization of specimens

The specimens were initially cleaned and hydroxylated by immersing them in piranha solution (3:1 concentrated sulfuric acid and 30% hydrogen peroxide) for 1 min, followed by thorough rinsing with Milli-Q water. After air drying, the Ti6Al4V and Y-TZP discs were placed in a round-bottom flask equipped with a magnetic stirrer and soaked in a 5 vol% solution of 3-aminopropyldiisopropylethoxysilane (APDS) in anhydrous toluene. The silanization reaction was carried out under reflux at 120 °C for 3 h. Subsequently, the discs were rinsed with technical toluene, followed by Milli-Q water, and then left to dry overnight at room temperature.

### Biofunctionalization – Immobilization of fibronectin and laminin

To immobilize fibronectin or laminin on the silanized surfaces, a bis(sulfosuccinimidyl)-suberate (BS^3^) crosslinker was used. Silanized discs were incubated with a 0.25 mM BS^3^ solution in borate-buffered saline (BBS, pH 8.5) at 4 °C for 1 h under constant agitation. Following crosslinking, the discs were thoroughly rinsed with precooled BBS and then immersed in a protein solution containing 1 µg/mL of either fibronectin or laminin in BBS. Full-length fibronectin (human plasma–derived, Merck/Sigma-Aldrich, cat. no. 341635) and laminin-111 (laminin from human fibroblasts, Merck/Sigma-Aldrich, cat. no. L4544) were used for surface biofunctionalization. Full-length ECM proteins were selected to preserve native multivalent binding domains and integrin-mediated clustering as established in our previous immobilization protocol [31].

The protein incubation was conducted for 2 h at 4 °C with frequent shaking to facilitate covalent bonding of the proteins to the surface. To remove any unbound proteins, the discs were washed three times with precooled BBS. The nomenclature of the various specimens used in this study is detailed in Table 1.

**Table 1.** Nomenclature of the individual specimens.

Specimens	Sample description
Ti6Al4V	Untreated Ti6Al4V specimens
Ti6Al4V-FN	Ti6Al4V after activation with silanization and fibronectin
Ti6Al4V-LN	Ti6Al4V after activation with silanization and laminin
Y-TZP	Untreated Y-TZP specimens
Y-TZP-FN	Y-TZP after activation with silanization and fibronectin
Y-TZP-LN	Y-TZP after activation with silanization and laminin

### Experimental setup for *in vitro* biofilm formation

Saliva collection and experimental procedures were approved by the RWTH Aachen University ethics committee (EK 165/10) and performed with informed consent from donors. Unstimulated saliva was simultaneously collected from ten healthy donors (five females and five males). Donors were instructed to refrain from brushing their teeth for 24 h and to abstain from consuming food or drink for at least 4 h prior to providing saliva samples in the morning. The collected saliva samples were pooled and mixed with an anoxic 60% glycerol solution containing 37 g/L brain heart infusion (BHI) broth in a 1:1 ratio, yielding a final concentration of 30% glycerol. The saliva stock was stored at –80 °C in 2 mL aliquots until use. A modified BHI medium was utilized as a substrate for microcosm biofilm cultivation. The medium was prepared under anoxic conditions and supplemented with 0.2% sucrose, 0.5% yeast extract, 1 µg/mL resazurin, 0.005 g/L hemin, 0.01 g/L menadione, 0.5 g/L L-cysteine hydrochloride, 0.87 g/L arginine, and 50 mM 1,4-piperazinediethanesulfonic acid (PIPES) buffer in addition to the standard BHI components.

Sucrose served as an additional substrate to efficiently promote biofilm formation [32,33]. Resazurin was added as a redox indicator to ensure anoxic conditions within the medium [34,35]. Hemin and menadione were included as essential compounds for the growth of fastidious oral bacteria [36,37]. L-cysteine hydrochloride was utilized to reduce the redox potential of the growth medium [35,38]. PIPES buffer was used to maintain a stable pH, which is essential for studying biofilms and the oral microbiome [32].

The pH of the medium was adjusted to 7.6. The saliva stock was diluted in modified BHI medium at a ratio of 1:50. This dilution was chosen based on previous work to ensure optimal microbial diversity while avoiding overgrowth during incubation, thereby maintaining physiological relevance in the static biofilm model [32,39]. The specimens were inoculated with the solution in 24-well plates under anaerobic conditions. Biofilm formation was monitored after 1, 2, 3, and 4 days of incubation.

### Crystal violet analysis

For qualitative assessment of biofilm formation, biofilm-covered discs were transferred to a new 24-well plate, fixed with 800 µL of 4% paraformaldehyde (PFA) for 10 min, rinsed with 800 µL of distilled water and stained with 800 µL of 0.1% crystal violet (CV) solution for 20 min. Excess dye was removed by a final rinse with distilled water (800 µL). Subsequently, the discs were air-dried overnight. Crystal violet staining was used exclusively for visual inspection of total biofilm coverage, while quantification of viable bacterial cells was performed separately using impedance flow cytometry.

### Scanning electron microscopic (SEM) analysis for observation of biofilm morphology on specimens

SEM was used to examine the biofilm structure and evaluate the suppression effects of biofunctionalization. The biofilm-covered specimens were transferred to a new 24-well plate and washed with 800 µL of PBS. The developed biofilm layer was then fixed with 2.5% glutaraldehyde for at least 1 h at room temperature before being stored at 4 °C. Subsequently, the specimens were rinsed with 0.1 M Soerensen's phosphate buffer for 15 min. Following rinsing, the specimens underwent a dehydration process using a gradient series of ethanol (EtOH): 10 min in 30% EtOH, 10 min in 50% EtOH, 10 min in 70% EtOH, 10 min in 90% EtOH and three consecutive 10-min exposures in 100% EtOH. After dehydration, the specimens were sputter-coated with a 10 nm gold-palladium layer and examined under SEM at an accelerating voltage of 10 kV. Three random locations on each disc were imaged to analyze biofilm morphology.

### Microcosm biofilm collection

Biofilm from three specimens of each group, which were not designated for crystal violet and SEM analyzes, was collected and disaggregated to obtain single cells for subsequent analyzes, including viable cell quantification, dead/live cell microscopic analysis and DNA extraction. The disaggregation process involved mechanical removal of the biofilm using 0.5 mL of anoxic phosphate-buffered saline (PBS) supplemented with peptone, followed by pipetting and vortexing for 30 s to further disrupt the biofilm structure. Subsequently, the samples were subjected to ultrasonication in cold water for 1 min, repeated twice, to ensure complete disaggregation of biofilm aggregates. This procedure facilitated the isolation of single cells, enabling accurate and reliable downstream analyzes.

### Viable cell quantification with impedance flow cytometry

Viable cell quantification was performed using impedance flow cytometry (IFC) with the BactoBox system (SBT Instruments, Denmark). Prior to sample analysis, the instrument was calibrated according to the manufacturer's instructions using calibration beads provided by the manufacturer. The conductivity was set between 1,600 and 2,100 µS/cm in the final samples. Bacterial cell samples were appropriately diluted to achieve concentrations ranging from 10,000 to 5,000,000 intact cells/mL.

### Dead/live cell microscopic analysis of biofilm

After 1, 2, 3 and 4 days of cultivation, biofilm-covered specimens were transferred to a fresh 24-well plate. A working solution of fluorescent stains was prepared by adding 3 μL of SYTO 9 stain and 3 μL of propidium iodide stain (Molecular Probes, Invitrogen Corp., Carlsbad, CA, USA) to 1 mL of PBS in a foil-covered container. About 200 μL of staining solution was gently applied to each biofilm sample to avoid disturbing the biofilm structure. The samples were then incubated for 30 min at room temperature, protected from light.

Following incubation, excess dye was removed, and each disc was gently scraped in 10 µL of sterilized distilled water to dislodge the stained biofilm. The specimens were subsequently examined using a high-performance fluorescence microscopy (FM) and contrast microscopy system (Leica DM6000B, Wetzlar, Germany). The excitation/emission was 488 nm/<550 nm for SYTO 9 with L5 filter and 555 nm/>550 nm for propidium iodide with N21 filter.

Three random images were captured from each disk. The percentage of dead bacteria was quantified using Fiji (ImageJ, US National Institutes of Health, Bethesda, Maryland, USA). The image type of 8-bit was chosen for the analysis. Fiji macro was downloaded from Allevi [40], and installed accordingly.

### DNA extraction

Genomic DNA (gDNA) extraction was performed using the Monarch Genomic DNA Purification Kit (NEB, USA) following the manufacturer's protocol. Saliva samples, planktonic bacteria obtained from saliva cultured in BHI medium without specimens (hereafter abbreviated as WS), and biofilm-derived single cells were centrifuged at 16,200 × g for 2 min and subsequently resuspended in cold PBS. All samples were vortexed and centrifuged at 16,200 × g for 1 min. Supernatant was removed from each sample and pellet in ~100 µL PBS was kept in the tube. Subsequently, each sample was vortexed and added with 10 µL proteinase K and 10 µL RNase A. The sample was briefly vortexed, added with 100 µL cell lysis buffer, vortexed again and incubated at 56 °C for 30 min in a thermal mixer with agitation at 1,400 rpm. The resulting gDNA was then bound to the purification column provided in the kit. After binding, the column was washed to remove impurities and contaminants, and the pure gDNA was eluted using preheated elution buffer. Concentration and purity of the extracted gDNA were checked using a nanodrop spectrophotometer (Nanodrop One, Thermo Scientific, Germany).

## 16S rRNA sequencing pipeline

The quality of the isolated genomic DNA (gDNA) samples was assessed using the TapeStation assay (Agilent), and their concentrations were quantified with the Quantus fluorometer (Promega, Germany). The starting concentrations of all samples are adjusted prior to library construction. Sequencing libraries targeting the V3/V4 region of the 16S rRNA gene were prepared using the Quick 16S NGS Library Prep Kit (Zymo Research Europe GmbH, Germany). The prepared libraries were pooled equimolarly and sequenced using the MiSeq Reagent Kit v3 (600 cycles) on an Illumina MiSeq platform (Illumina, USA).

The 16S rRNA sequencing data were analyzed using a standardized and reproducible workflow. FASTQ files were generated with bcl2fastq (Illumina, USA). To ensure reproducibility, samples were processed using the publicly available nf-core/ampliseq pipeline version 2.6.1 [41]. The pipeline was executed with Nextflow v23.04.1 [42] and Docker 20.10.12 [43] using the minimal command. In brief, the sequencing reads underwent quality control with FastQC and were trimmed using Cutadapt. Amplicon sequence variants were inferred with DADA2, applying a low abundance filter of 10 copies. Taxonomic classifications were performed using DADA2 and QIIME2 with the SILVA 138 reference taxonomy [44].

## Data analysis

Downstream analyzes (Shannon diversity index, Bray–Curtis dissimilarity index, Aitchison distance, and SparCC) and visualizations were conducted using customized scripts and Bioconductor packages in R (version 4.2.2). Briefly, data from all samples were rarefied to 90% of the minimum sequencing depth to normalize read counts, followed by calculating Shannon diversity indices using the phyloseq package [45] in R. Bray–Curtis distances were also computed for all samples by applying the same package. In addition to Bray–Curtis dissimilarity analysis, Aitchison distance was applied to account for the compositional nature of the microbiome data. The dataset was transformed using centered log-ratio (CLR) transformation after filtering out low-abundance taxa present in fewer than 10% of samples and Euclidean distances were calculated on the CLR-transformed data to obtain Aitchison distances. For SparCC network analysis, taxa were first filtered based on prevalence (0.1) and relative abundance (0.1%) thresholds to remove low-abundance and infrequently detected features and then were processed using the phyloseq and SpiecEasi [46] packages in R. The raw sequencing data generated in this study are publicly available in the NCBI Sequence Read Archive (SRA) under BioProject accession number PRJNA1211561.

## Short-chain fatty acids (SCFAs) assay

The production of short-chain fatty acids (SCFAs) was assessed in real time from the in vitro system and post-formation from the biofilm alone. Real-time SCFA production was determined by collecting the biofilm-spent medium from the last medium refreshment before biofilm harvesting [47]. For post-formation SCFA production capacity, biofilm-covered specimens were transferred to a fresh 24-well plate containing 0.5 mL per well of buffered peptone water supplemented with 0.2% sucrose. The plates were then incubated anaerobically for 3 h at 37 °C, and the supernatant was subsequently collected for SCFA analysis [32].

Both real-time and post-formation SCFA productions were analyzed using an Ultimate 3000 High-Performance Liquid Chromatography (HPLC) system (Thermo Scientific, Waltham, MA, USA). The mobile phase consisted of 5 mM sulfuric acid (H₂SO₄) at a flow rate of 0.8 mL/min. Prior to injection, samples were centrifuged and filtered to remove particulates. Separation was achieved using a Metab-AAC column (Ion Exchange, 300 × 7.8 mm, 10 µm particle size; ISERA GmbH, Düren, Germany) maintained in a column oven at 60 °C. Detection was performed using a refractive index detector for acetic acid, butyric acid and formic acid, and a UV detector set at 210 nm for lactic acid.

## Statistical analysis

Experiments were performed in triplicates, and results were analyzed using one-way ANOVA with Tukey's multiple comparison test. Differences were considered statistically significant if *p* < 0.05.

## Results

### Early biofilm formation on biofunctionalized Ti6Al4V and Y-TZP surfaces

**Figure 1.**
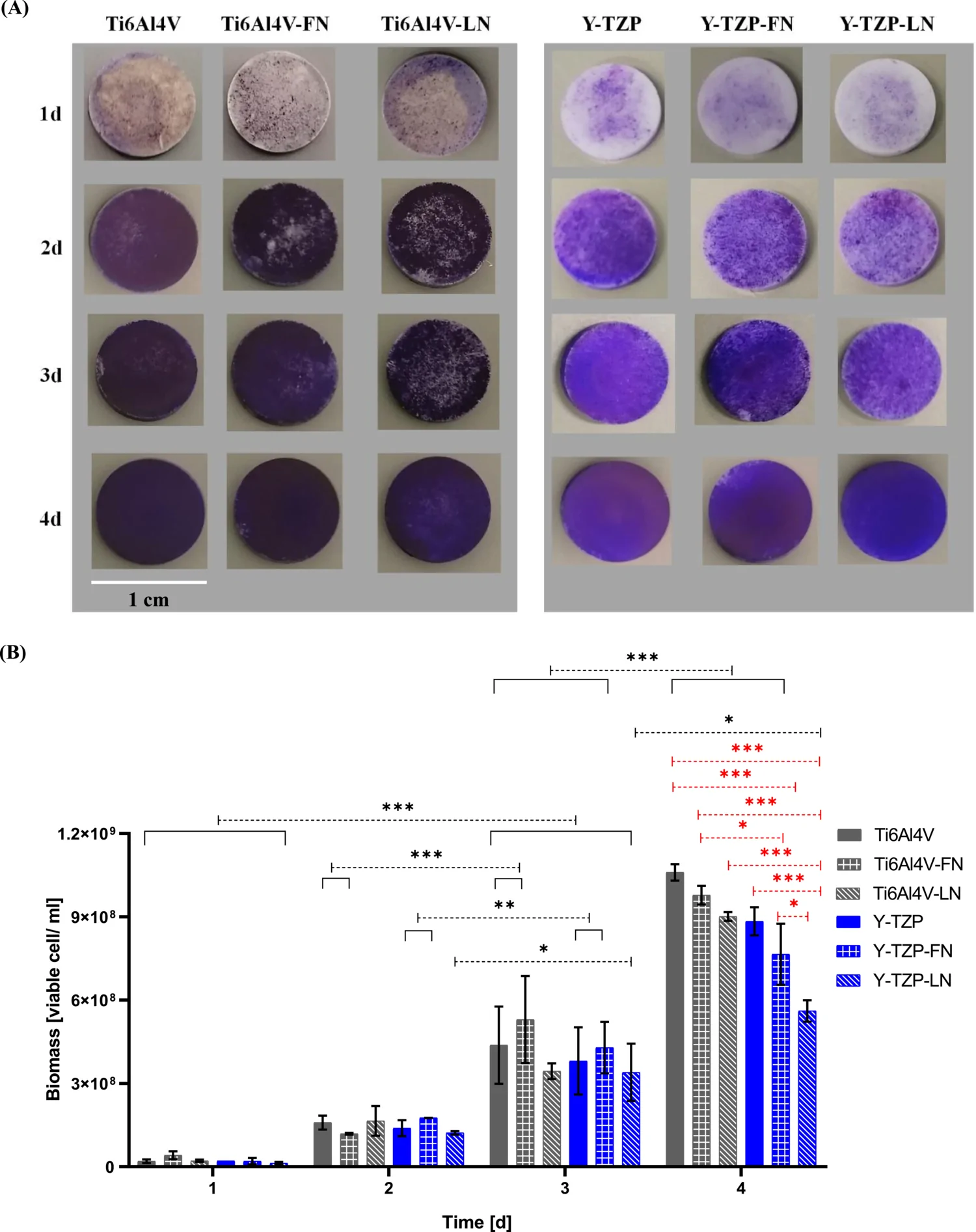
In vitro biofilm formation on control (Ti6Al4V, Y-TZP) and biofunctionalized surfaces (Ti6Al4V-FN, Ti6Al4V-LN, Y-TZP-FN, Y-TZP-LN). (A) Crystal violet staining showed progressive biofilm coverage with nearly complete surface colonization on day 4. (B) Impedance flow cytometry (IFC) revealed a time-dependent increase in viable biomass, with Y-TZP-LN showing the lowest values on day 4, significantly lower than all other groups. Error bars show ±  standard error of the mean (*n* = 3). One-way ANOVA with Tukey's test; *p < 0.05, **p < 0.01, ***p < 0.001. Black asterisks: differences over time within groups; red asterisks: differences between groups at the same time point.

Crystal violet staining revealed a progressive increase in biofilm coverage on all specimen groups, with partial colonization during the first three days and nearly complete surface coverage on day 4 (Figure 1A). Early visual differences between coatings were subtle, although LN-coated specimens were covered more slowly by biofilm than unmodified or FN-coated controls.

Impedance flow cytometry (IFC) confirmed a gradual increase in viable cell density between days 1 and 3, followed by a further increase to day 4 (Figure 1B). On day 3, differences between coatings were minor; however, on day 4, Y-TZP-LN specimens exhibited the lowest viable biomass (5.61 × 10^8^ cells/mL), significantly reduced compared to all other groups. Ti6Al4V surfaces generally supported higher viable cell numbers than Y-TZP, although these differences did not reach statistical significance.

**Figure 2.**
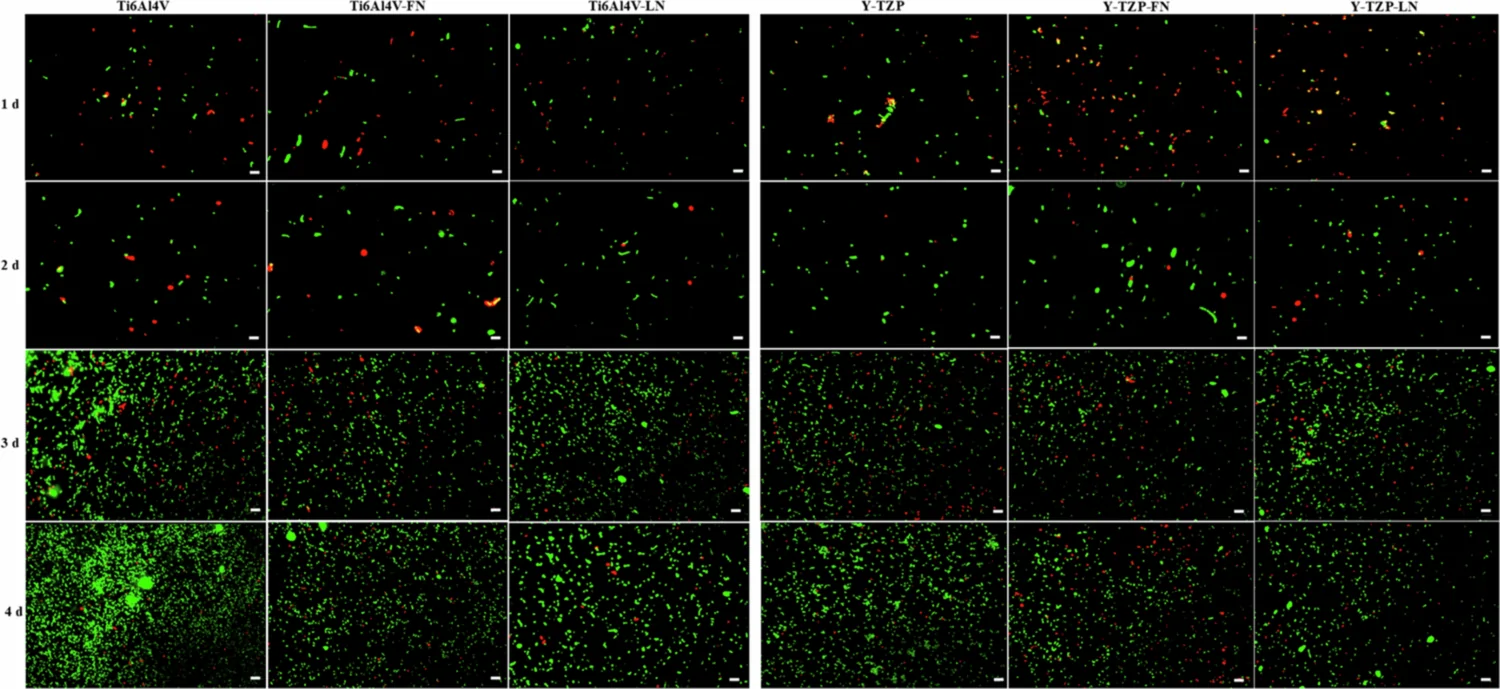
Fluorescence microscopy of live (green, SYTO 9) and dead (red, propidium iodide) bacterial cells on Ti6Al4V and Y-TZP specimens, unmodified or coated with fibronectin (FN) or laminin (LN), after 1–4 days of incubation. Images show progressive bacterial colonization over time. Scale bars (10 µm) are shown in all panels. All images were acquired at 630×  magnification. Experiments were performed in triplicate (*n* = 3).

**Figure 3.**
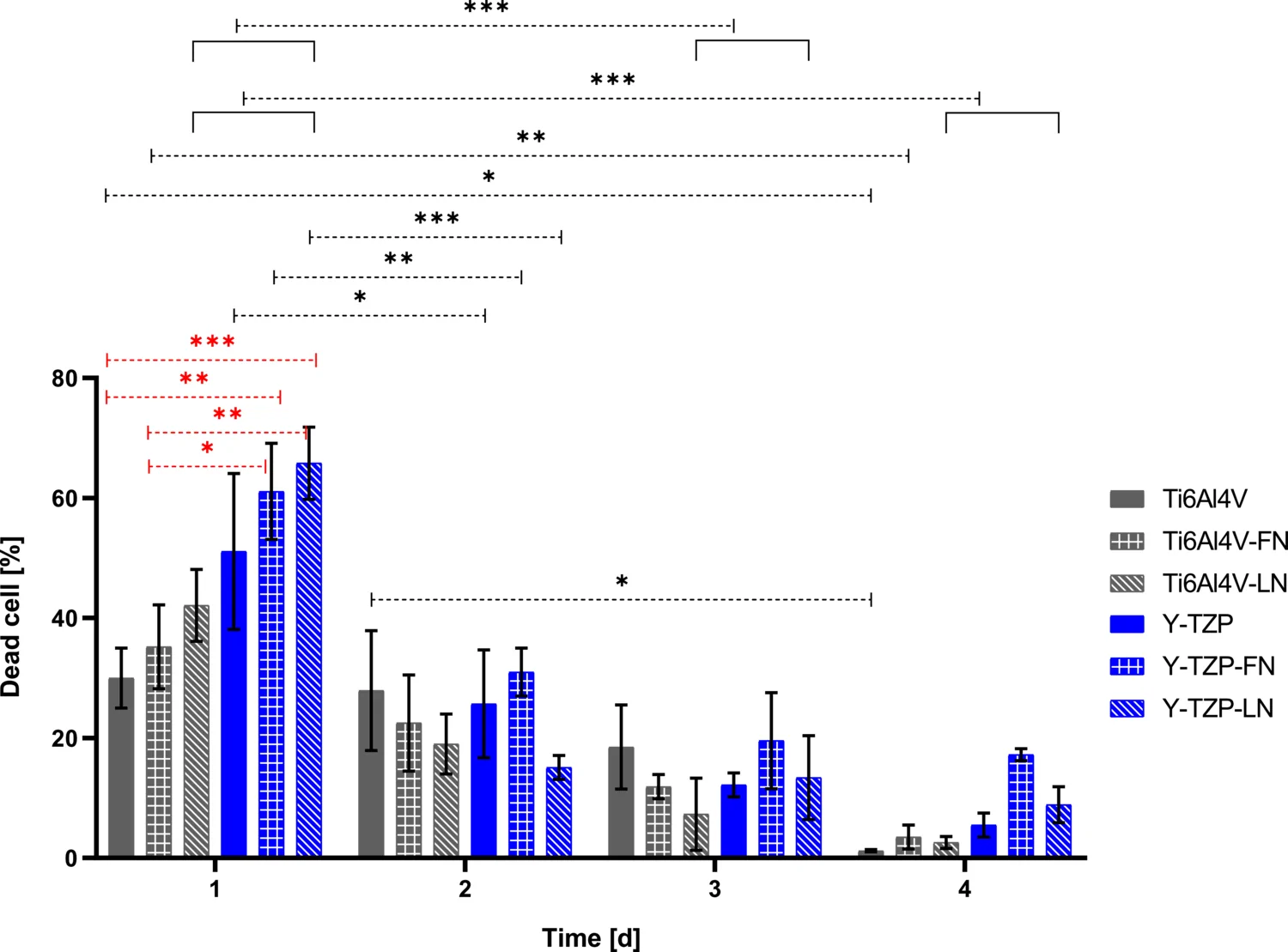
Percentage of dead cells on Ti6Al4V and Y-TZP specimens, unmodified or coated with fibronectin (FN) or laminin (LN), over 1–4 days of incubation. Cell death peaked on day 1, particularly for Y-TZP coatings and declined steadily thereafter, reaching minimal levels on day 4. Error bars show ± standard error of the mean (*n* = 3). One-way ANOVA with Tukey's test; *p < 0.05, **p < 0.01, ***p < 0.001. Black asterisks: differences over time within groups; red asterisks: differences between groups at the same time point.

Fluorescence microscopy (FM) provided complementary insights into bacterial viability (Figure 2). During the first two days, numerous dead cells (red) were visible across all groups, particularly on Y-TZP specimens. Quantitative Fiji analysis confirmed that the highest proportions of dead cells occurred on day 1, with Y-TZP-LN reaching up to 66% and Y-TZP-FN also showing elevated levels compared to unmodified Y-TZP (Figure 3). Ti6Al4V specimens followed a similar trend, with LN- and FN-coated surfaces showing slightly higher early dead-cell fractions than the unmodified control. Thereafter, the proportion of dead cells declined steadily in all groups, resulting in predominantly viable biofilms (green) by day 4. This decline was most pronounced on Ti6Al4V specimens, consistent with their higher viable biomass in IFC analysis.

**Figure 4.**
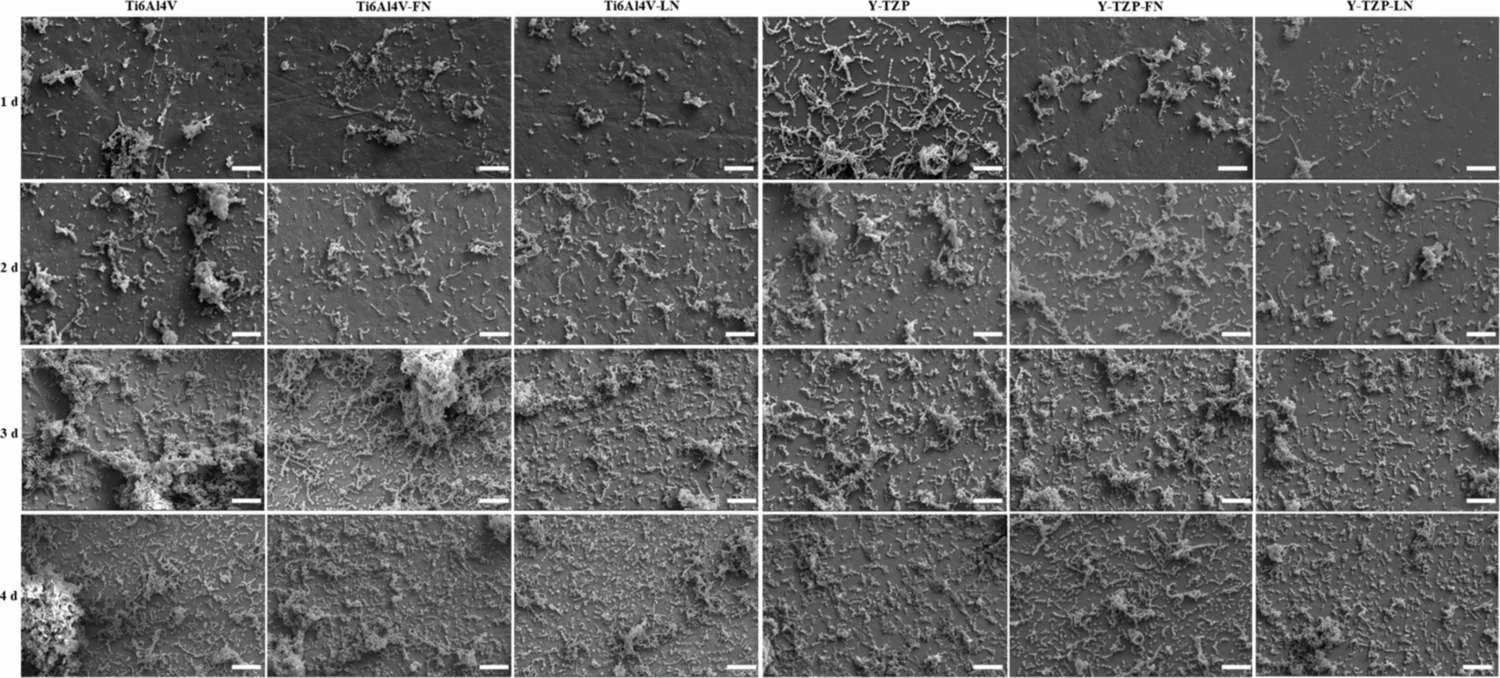
Scanning electron microscopy (SEM) of biofilms on Ti6Al4V and Y-TZP specimens, unmodified or coated with fibronectin (FN) or laminin (LN), after 1–4 days of incubation. Images show the progression of biofilm development and extracellular polymeric substance (EPS) formation across surfaces. Scale bars (10 µm) are shown in all panels. All images were acquired at 2,500×  magnification. Experiments were conducted in triplicates (*n* = 3).

SEM imaging qualitatively illustrated the morphological progression of biofilm development (Figure 4). Across all groups, bacterial attachment and extracellular polymeric substance (EPS) accumulation increased progressively, leading to dense confluent biofilms on day 4. On Ti6Al4V, FN- and LN-coated surfaces differed little beyond the first day, when FN appeared slightly denser than LN. In contrast, Y-TZP-LN surfaces displayed markedly reduced bacterial attachment and minimal EPS deposits after one day, indicating delayed early colonization. These ultrastructural findings corroborated the quantitative analyzes, reinforcing Y-TZP-LN as the surface condition most resistant to early biofilm establishment.

Taken together, the complementary methods consistently demonstrated that while all surfaces eventually developed dense biofilms on day 4, laminin coating on Y-TZP significantly delayed early biofilm maturation and reduced viable biomass, identifying it as the most effective strategy to mitigate bacterial colonization among the tested conditions.

### Microbial profiles and key genus dynamics on biofunctionalized specimens

**Figure 5.**
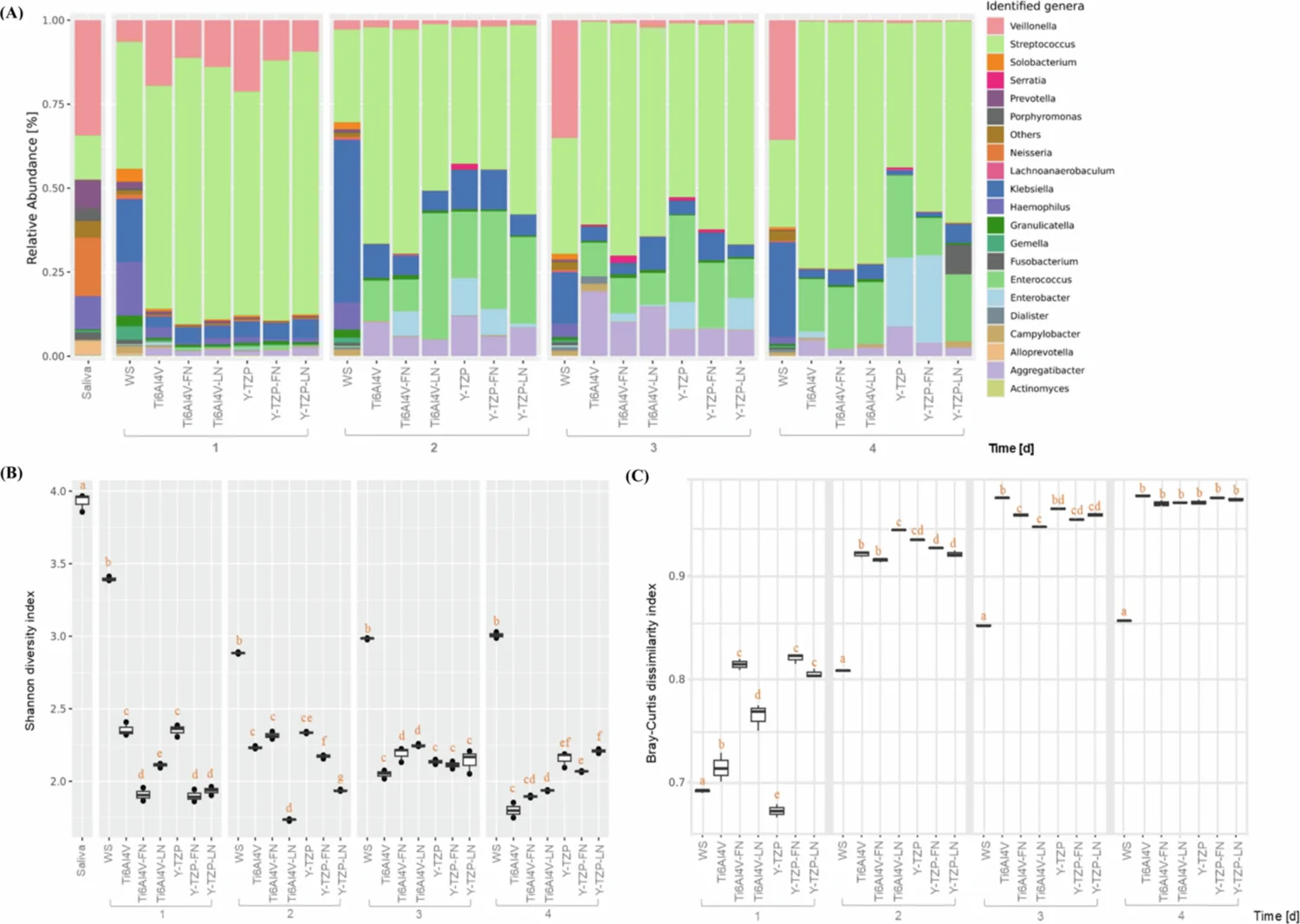
(A) Relative microbial abundance profiles, (B) Shannon diversity index and (C) Bray–Curtis dissimilarity index of the saliva inoculum, in vitro planktonic bacteria without specimens (WS), and oral microcosm biofilms formed on control (Ti6Al4V and Y-TZP) and biofunctionalized (Ti6Al4V-FN, Ti6Al4V-LN, Y-TZP-FN, and Y-TZP-LN) surfaces over the incubation period. Statistical analysis was performed using one-way ANOVA with Tukey's multiple comparison test; different letters indicate significant differences at *p* < 0.05. Error bars show ± standard error of the mean (*n* = 3).

Analysis of the saliva inoculum revealed a diverse microbial composition dominated by *Veillonella* (33%), *Neisseria* (17%), *Streptococcus* (13%), and *Haemophilus* (10%) (Figure 5A). When cultured without specimens (WS), planktonic communities were initially dominated by *Streptococcus* and gradually shifted toward *Klebsiella* and *Veillonella* during incubation. Similar dominant genera were identified in biofilms formed on both non-modified and biofunctionalized surfaces, with *Streptococcus* remaining prevalent throughout.

In this study, the Shannon diversity index was used to assess *α* diversity within individual biofilm samples (*n* = 3) grown on control (Ti6Al4V and Y-TZP) and biofunctionalized (Ti6Al4V-FN, Ti6Al4V-LN, Y-TZP-FN, and Y-TZP-LN) surfaces as well as the native saliva inoculum and planktonic bacteria (WS) samples at each incubation time. The most pronounced delay was observed on day 1 in biofilms grown on biofunctionalized surfaces, suggesting that these coatings imposed an early selective pressure on microbial adhesion and colonization (Figure 5B).

Complementary Bray–Curtis dissimilarity analysis (Figure 5C) revealed that biofilms formed on biofunctionalized specimens were significantly more dissimilar from the saliva inoculum than those on WS or non-modified surfaces, particularly on day 1. This early divergence indicated that the ECM protein coatings (especially laminin) not only altered microbial adhesion but also redirected the ecological trajectory of biofilm development.

**Figure 6.**
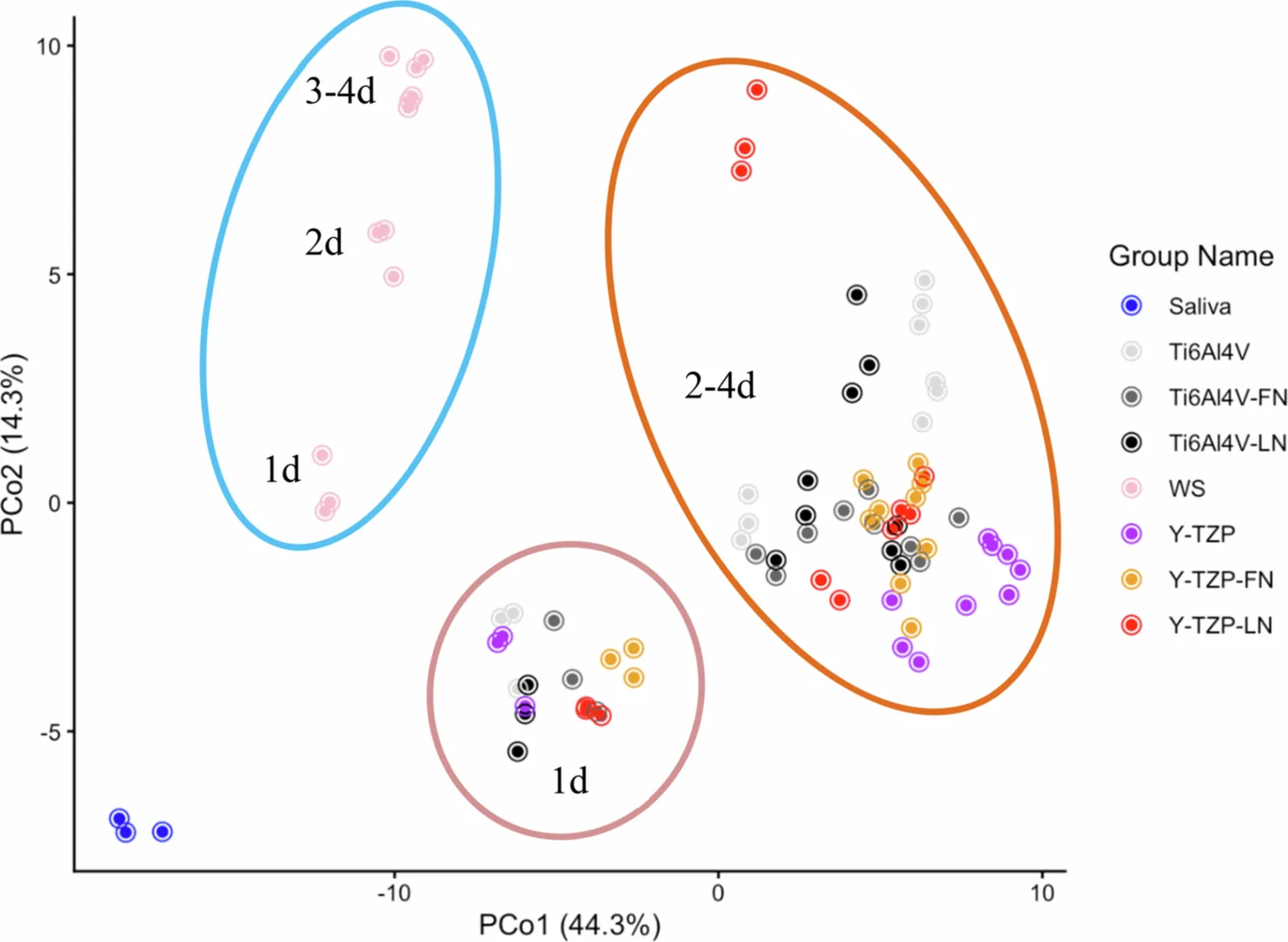
Aitchison distance of the saliva inoculum, in vitro planktonic bacteria without specimens (WS) and oral microcosm biofilms formed on control (Ti6Al4V and Y-TZP) and biofunctionalized (Ti6Al4V-FN, Ti6Al4V-LN, Y-TZP-FN, and Y-TZP-LN) surfaces.

The Aitchison distance-based ordination revealed distinct clustering patterns primarily along the PCo1 (44.3% explained variance), separating day 1 from day 2–4 biofilm samples, consistent with a pronounced shift in community composition during initial colonization. The saliva inoculum was clearly separated from both biofilm and planktonic bacteria (WS) samples, mainly along PCo1 and to a lesser extent along PCo2 (14.3%), suggesting divergence from the original microbial community. WS samples occupied an intermediate position between saliva and biofilms while remaining distinct from both, indicating differences between planktonic and surface-associated microbial communities (Figure 6).

Comparison of identified genera (Table 2) showed that planktonic bacteria (WS) samples retained a high overlap with saliva (91%), whereas surface-associated biofilms displayed substantially lower similarity (51–64%). The reduced overlap on fibronectin- and laminin-functionalized specimens (~58%) suggests that certain taxa preferentially remained planktonic, while the coatings selectively influenced which bacterial genera successfully attached and persisted during initial biofilm development.

**Table 2.** The overlap of identified genera in the compared conditions, based on relative abundance of data from 16S rRNA sequencing.

	∑ of genera	Group name	Overlap to saliva (%)				
			Day 1	Day 2	Day 3	Day 4	Overall
Saliva	55	WS	86	73	75	67	91
		Ti6Al4V	56	44	29	27	64
		Ti6Al4V-FN	55	50	35	33	58
		Ti6Al4V-LN	53	42	35	35	58
		Y-TZP	60	40	35	20	64
		Y-TZP-FN	51	38	35	27	51
		Y-TZP-LN	53	40	37	37	58

**Figure 7.**
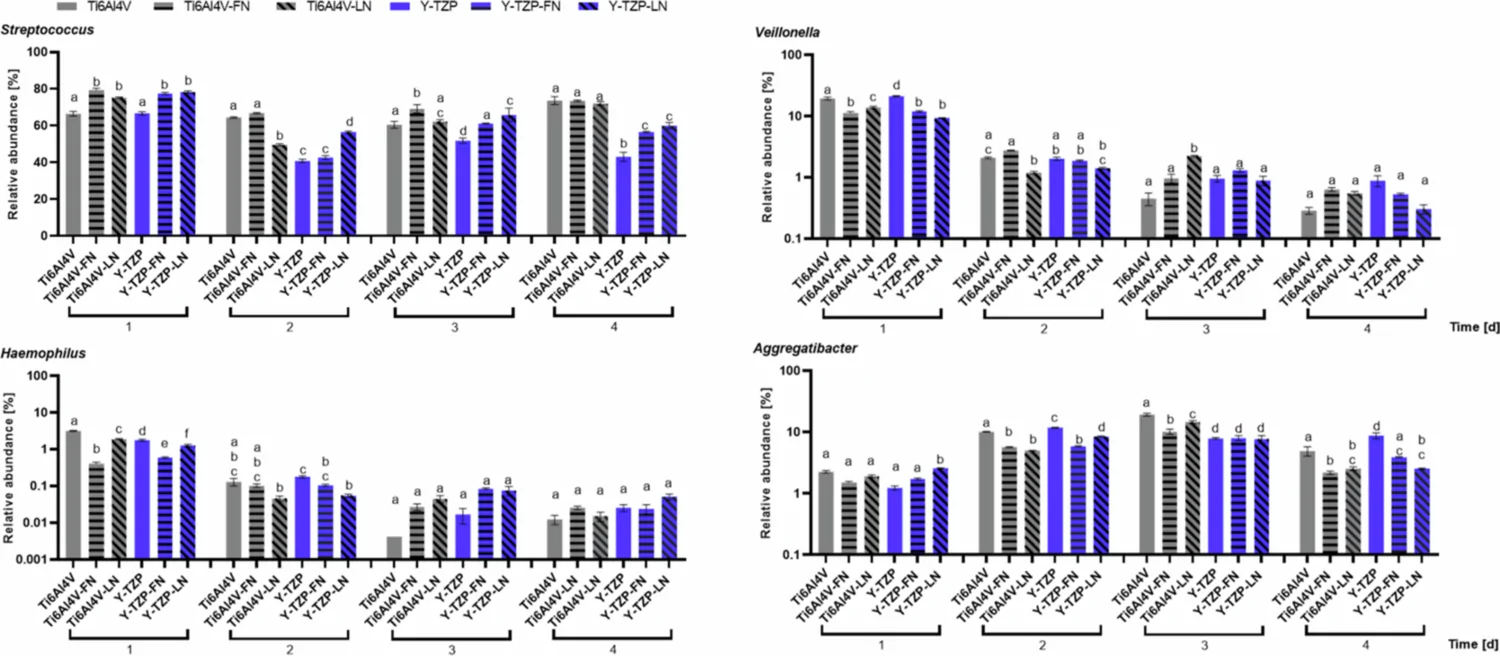
Relative abundance (%) of selected bacterial genera (*Streptococcus*, *Haemophilus*, *Veillonella* and *Aggregatibacter*) in oral microcosm biofilms formed on non-biofunctionalized (Ti6Al4V and Y-TZP) and biofunctionalized (Ti6Al4V-FN, Ti6Al4V-LN, Y-TZP-FN, and Y-TZP-LN) specimens over the incubation period. Statistical analysis was performed using one-way ANOVA with Tukey's multiple comparison test; different letters indicate significant differences at *p* < 0.05. Error bars show  ± standard error of the mean (*n* = 3).

To further elucidate which taxa contributed to these compositional shifts, the relative abundance of four key oral genera—*Streptococcus*, *Haemophilus*, *Veillonella,* and *Aggregatibacter*—was analyzed separately (Figure 7). Among these, *Veillonella*, a bridging colonizer that metabolizes lactate from *Streptococcus*, was markedly reduced on all biofunctionalized surfaces compared with non-modified ones. The lowest relative abundance occurred on Y-TZP-LN (9%) after one day of incubation. This transient suppression coincided with the delayed biofilm biomass accumulation observed in IFC and crystal violet analyzes. In contrast, Streptococcus remained relatively stable across all surfaces, while *Haemophilus* and *Aggregatibacter* showed only minor variations without consistent trends. Together, these results suggest that laminin coatings may transiently disrupt early cross-feeding interactions between *Streptococcus* and *Veillonella*, thereby postponing the transition from initial adhesion to a mature, structured biofilm.

**Figure 8.**
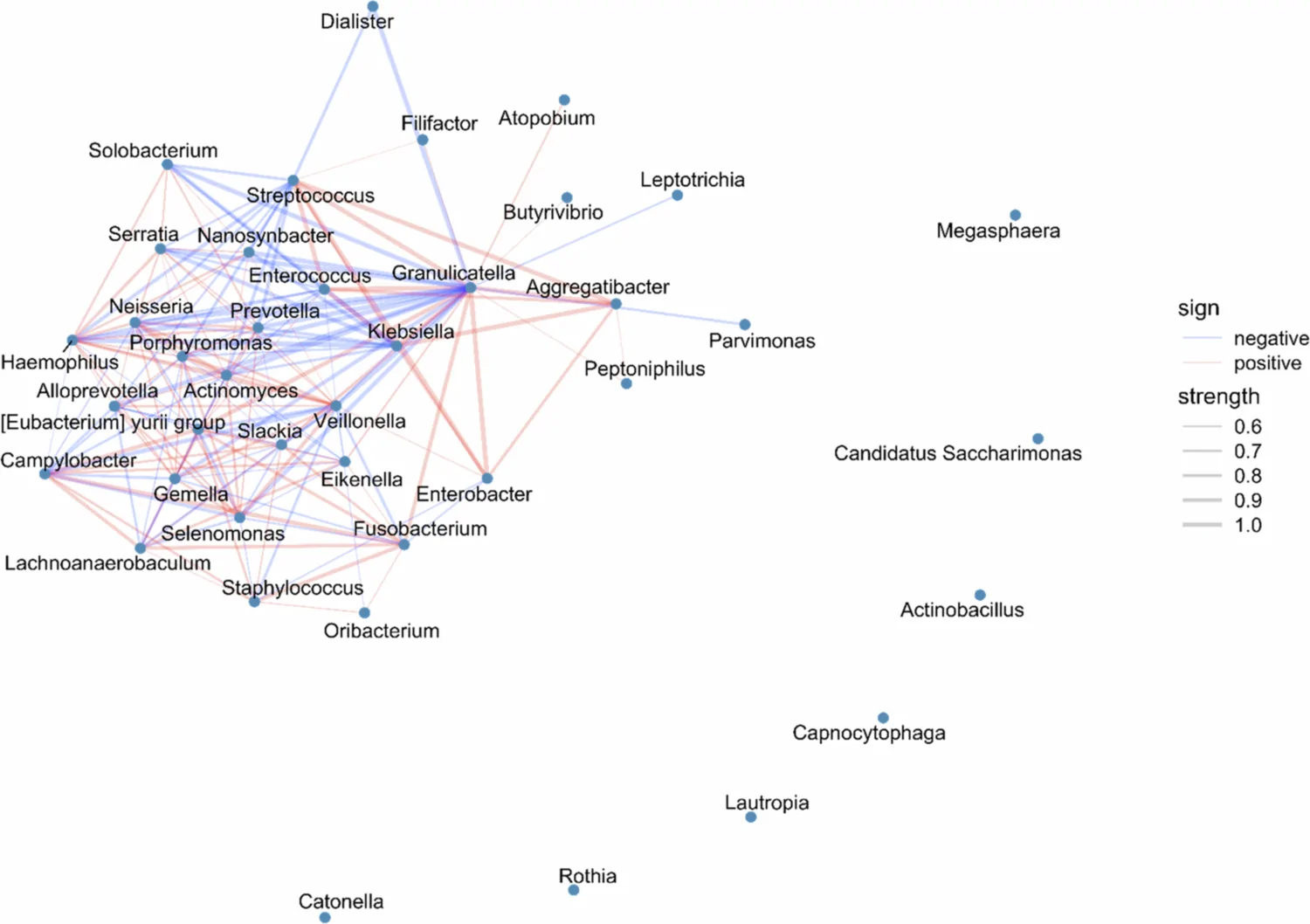
Bacterial association networks of biofilm on Y-TZP-LN constructed using SparCC correlation coefficients for compositional data retaining ASVs with at least 0.1%. A correlation threshold of 0.5 was applied in visualization. Positive correlations were visualized as red lines and negative correlations as blue lines.

SparCC-based association network analysis (Figure 8) was performed to assess potential changes in microbial co-occurrence patterns associated with laminin-functionalized Y-TZP (Y-TZP-LN). Biofilms on unmodified Y-TZP exhibited a dense and highly interconnected network structure (Appendix Figure 1). On Y-TZP-FN, *Streptococcus* remained broadly connected, and *Veillonella* continued to show integration within the network (Appendix Figure 2). Their associations with secondary colonizers such as *Aggregatibacter* and *Prevotella* were preserved, indicating only moderate reorganization of interspecies relationships (Appendix Figure 2). Notably, a direct association between *Streptococcus* and *Veillonella* was observed in biofilms on Y-TZP and Y-TZP-FN, whereas this connection was absent in the network derived from Y-TZP-LN (Figure 8).

**Figure 9.**
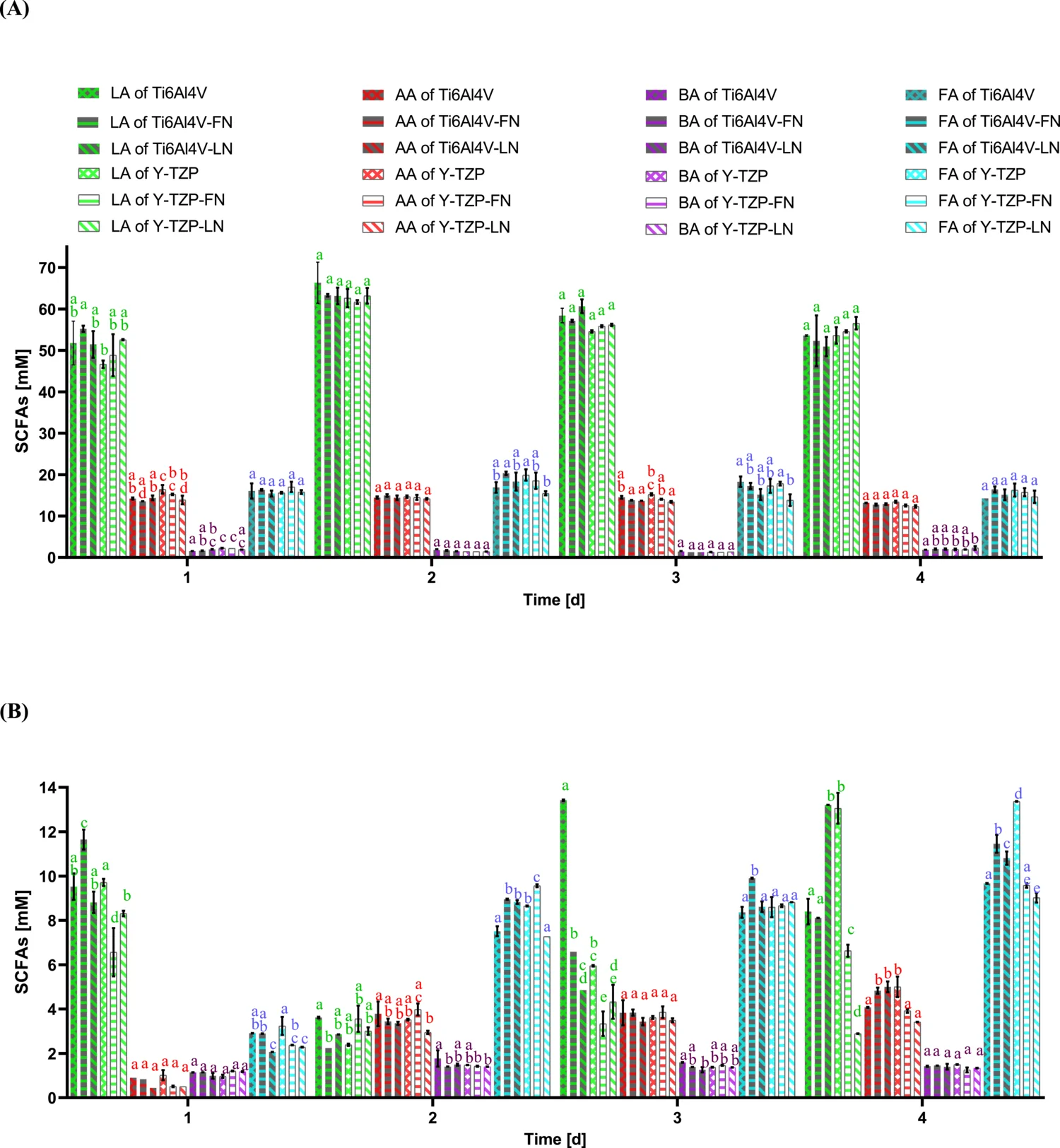
Short-chain fatty acid (SCFA) production, namely lactic acid (LA), acetic acid (AA), butyric acid (BA) and formic acid (FA), measured from (A) real-time culture medium and (B) post-formation biofilms on control (Ti6Al4V and Y-TZP) and biofunctionalized (Ti6Al4V-FN, Ti6Al4V-LN, Y-TZP-FN, and Y-TZP-LN) surfaces. Statistical analysis was performed using one-way ANOVA with Tukey's multiple comparison test; different letters indicate significant differences at *p* < 0.05. Error bars show ± standard error of the mean (*n* = 3).

### Biofilm dynamics and short-chain fatty acid (SCFA) production

To assess not only the microbial composition but also the functional activity of biofilms, short-chain fatty acid (SCFA) production was analyzed under two complementary conditions: real-time SCFA production, reflecting metabolic activity during biofilm growth and post-formation SCFA production, representing the metabolic potential of the mature biofilm itself (Figure 9). Together, these analyzes provide insight into how biofunctionalized surfaces influence both the dynamics and functional maturation of the biofilm community.

The culture medium was adjusted to pH 7.6 and refreshed twice during incubation, resulting in a final average pH of 6.08 ± 0.37. This decrease reflected active SCFA production and indicated ongoing microbial metabolism within the microcosm biofilm.

As shown in Figure 9A, real-time SCFA production across all specimens was dominated by lactic acid (≈63%), followed by formic acid (≈19%) and acetic acid (≈16%), confirming that lactic acid was the major metabolic byproduct during early biofilm development. From day 1 to 3, biofilms on Ti6Al4V surfaces—both non-functionalized and biofunctionalized—produced higher lactic acid levels than those on Y-TZP surfaces, suggesting a higher abundance and activity of lactic acid–producing bacteria on titanium.

Post-formation SCFA profiles (Figure 9B) revealed distinct temporal and surface-dependent metabolic shifts. Lactic acid concentrations peaked on day 1 and declined significantly on day 2, while acetic and formic acid levels increased, reflecting a metabolic transition characteristic of biofilm maturation. On day 4, Y-TZP-LN surfaces exhibited the lowest concentrations of both lactic and formic acids among all groups. This delayed biofilm formation likely corresponds to decreased abundance or activity of lactic acid–producing bacteria (LABs), excluding *Streptococcus*, which remained relatively stable across all surfaces. The concurrently reduced *Veillonella* abundance (Figure 7)—a key lactate-utilizing genus—offers a plausible explanation for diminished formic acid levels, as this genus converts lactate into SCFAs such as acetate and formate through syntrophic cross-feeding.

Together, these findings indicate that biofunctionalized surfaces not only delay microbial adhesion and total biomass but also interfere with cooperative metabolic networks within the biofilm, thereby attenuating the transition from early fermentative to mature syntrophic metabolism. These metabolic insights complement the compositional findings and suggest that surface biofunctionalization, particularly with laminin, may modulate both the ecological and metabolic trajectories of biofilm development—potentially contributing to a more host-compatible microbial interface.

## Discussion

### Overview and relation to previous work

Dental implant longevity depends not only on successful osseointegration but also on the establishment of a stable soft-tissue seal that limits bacterial penetration at the transmucosal interface. Surface biofunctionalization with extracellular matrix (ECM) proteins such as fibronectin (FN) and laminin (LN) has therefore been proposed to enhance host–implant interactions at this critical interface.

In our previous work [30], we established covalently immobilized ECM coatings on titanium alloy (Ti6Al4V) and yttria-stabilized zirconia (Y-TZP) and demonstrated their cytocompatibility, structural stability and ability to promote gingival fibroblast adhesion and integrin expression. These studies provided a robust foundation for evaluating ECM-based surface modifications under biologically relevant conditions.

Previous studies by other groups have shown that laminin-332-functionalized zirconia surfaces enhance epithelial cell adhesion and viability by improving surface bioactivity and protein presentation [48]. However, these investigations focused primarily on surface chemistry and host-cell responses and did not address bacterial colonization or the behavior of complex oral biofilms.

Building on these foundations, the present study shifts the focus from host-cell interactions to microbial dynamics. Using a validated saliva-derived oral microcosm model [39], we investigated whether covalently immobilized ECM coatings can modulate early bacterial adhesion, community structure and metabolic activity during the initial stages of oral biofilm formation.

### Delayed biofilm formation and mechanistic interpretation

Our findings demonstrate that ECM coatings influence early oral biofilm formation in a material- and protein-dependent manner. The strongest effects—manifested as reduced viable biomass , delayed extracellular polymeric substance (EPS) accumulation , and suppressed short-chain fatty acid (SCFA) production—were observed on laminin-functionalized Y-TZP, whereas fibronectin-coated and titanium-based specimens showed weaker modulation.

These results indicate that ECM-based coatings do not act as bactericidal barriers but instead transiently steer early biofilm development through a balance of surface chemistry and biological selectivity, consistent with the concept of biological steering [5,26]. Viable cell counts on Y-TZP-LN were lowest on day 4, suggesting that the modulatory effect persists throughout the observed colonization period. Whether such early interference can translate into long-term clinical benefit remains to be investigated.

The observed antibiofilm effects on laminin-functionalized Y-TZP surfaces likely arise from a combination of physicochemical and biological mechanisms. Increased surface hydrophilicity and surface energy reduce nonspecific bacterial adhesion by weakening hydrophobic interactions, particularly on smooth submicron surfaces [22,49]. Beyond these passive effects, ECM proteins provide defined receptor-binding motifs that favor host-cell attachment while competing with bacterial adhesins for surface binding sites.

Laminin, in particular, engages integrin receptors on gingival fibroblasts and epithelial cells and may plausibly act as a molecular decoy during early colonization, potentially hindering bacterial settlement without exerting bactericidal effects. In contrast, fibronectin-coated surfaces primarily enhance fibroblast adhesion and wound-healing responses via α5β1-integrin signaling [29,50], but lack direct antimicrobial or strong antibiofilm activity. This distinction is consistent with our findings, in which fibronectin coatings supported cytocompatibility yet showed only modest modulation of early biofilm development.

The basement-membrane origin of laminin further supports its role as a biologically selective interface. By mimicking epithelial attachment structures, laminin coatings may transiently favor host-cell recruitment while delaying bacterial adhesion, thereby influencing the early ‘race for the surface’. Notably, other laminin isoforms such as laminin-332 engage a distinct integrin receptor profile (α6β4/α3β1) and have been shown to enhance keratinocyte spreading and hemidesmosome formation [28,51]. For laminin-111, the isoform used in the present study, evidence regarding epithelial cell attachment appears substrate-dependent, with enhanced attachment reported on hydroxyapatite/polymer composites [52,53] but no significant effect observed on titanium alloy [54]; whether comparable effects apply to laminin-111-functionalized zirconia had, to our knowledge, not previously been investigated.

### Material–coating interactions

The antibiofilm performance of laminin coatings was strongly substrate dependent. Although both Ti6Al4V–LN and Y-TZP–LN delayed early biofilm development compared with unmodified controls, the decrease in viable cell numbers was markedly more pronounced on Y-TZP. It is acknowledged that protein adsorption to implant surfaces depends on several aspects, such as electrostatic interactions, wettability, topography, and charge [55]. Notably, external parameters including temperature and pH values of applied solutions also play a crucial role in protein adsorption kinetics [56]. Besides surface characteristics, protein properties (e.g. protein dimension, structural stability, zeta potential) are crucial [56]. The adsorption of fibronectin and albumin on titanium and zirconia was compared, in which a higher amount of the proteins was absorbed on negatively charged titanium [57]. Interestingly, more fibronectin compared to albumin was absorbed on both implant materials [57].

On the other hand, Y-TZP provides a chemically inert, negatively charged oxide surface that stabilizes protein conformation and favors uniform immobilization. In vivo, zirconia implants have been reported to exhibit a tighter mucosal seal and reduced epithelial down-growth compared with titanium, suggesting favorable soft-tissue interactions at the transmucosal interface [58]. In our study, ECM proteins were immobilized via a silane-based interfacial layer rather than directly interacting with the bulk material. Therefore, protein arrangement is primarily affected by crosslinking and the underlying silane layer. While Ti6Al4V and Y-TZP differ in surface reactivity and physicochemical properties, their impact on ECM protein formation is still limited through the organochemical modification step in between [59]. developed a bioinspired coating on titanium including biotinylated fibronectin. Within the study, they compared adsorbed fibronectin and biotinylated fibronectin to the titanium's surface. Importantly [59], found that nonspecific binding of fibronectin resulted in a globular conformation, which can cover biologically relevant binding sites within the protein structure. This could result in less human gingival cell adhesion and a higher risk of peri-implant disease.

Laminin immobilization on both Ti6Al4V and Y-TZP surfaces has previously been confirmed by immunofluorescence (Supplementary Information [31]), indicating successful coating on both substrates. However, this approach does not provide quantitative information on surface coverage or coupling efficiency. Therefore, potential differences in surface area, chemistry and physicochemical properties between titanium and zirconia may influence the actual amount and distribution of adsorbed protein. Future studies employing surface-sensitive techniques such as quartz crystal microbalance (QCM) or surface plasmon resonance (SPR) could provide further insight into material-dependent differences in protein adsorption and immobilization efficiency.

Furthermore, laminin-functionalized surfaces are particularly promising, including the underlying silane coating strategy, which has been extensively characterized in our previous studies using XPS and atomic force microscopy (AFM) [31]. We were able to show an increasing hydrophilic static contact angle by applying the BS^3^ crosslinker and fibronectin on the specimen's surface, as well as hydrolytic stability and enzymatic resistance of these bioinspired coatings [30]. These investigations confirmed successful formation of the functional silane layer and the covalent immobilization of ECM proteins such as fibronectin. Additionally, human gingival fibroblasts revealed enhanced expression of important adhesion molecules on laminin- and fibronectin-modified surfaces [31].

### Community composition and metabolic activity

Microbial and metabolic analyzes revealed that ECM coatings, particularly laminin-functionalized Y-TZP, significantly altered early oral biofilm formation. 16S rRNA sequencing showed that LN-functionalized Y-TZP surfaces developed a distinct microbial profile characterized by reduced *α*-diversity and increased Bray–Curtis dissimilarity relative to the saliva inoculum, indicating an early selective pressure on microbial adhesion and community assembly.

In saliva-derived microcosm models, early biofilms are typically dominated by *Streptococcus* and *Veillonella* species, which establish metabolic cooperation through lactate cross-feeding [39,60]. In the present study, this syntrophic interaction was markedly disrupted on LN-coated Y-TZP surfaces, where *Veillonella* abundance was reduced to approximately 9% on day 1. Since *Veillonella* relies on lactate produced by *Streptococcus* to generate acetate, propionate, and formate [61,62], this delayed biofilm formation suggests that laminin coatings attenuate early metabolic cooperation within the biofilm community.

SparCC-based network analysis revealed the absence of a direct association between *Streptococcus* and *Veillonella* on laminin-functionalized zirconia, indicating disruption of a key interaction axis in early biofilm development. This interaction is known to underpin metabolic cross-feeding, particularly through lactate exchange, which supports the transition from early colonizers to secondary colonizers, a more complex, and metabolically active biofilm. Its absence is therefore consistent with impaired microbial coordination and delayed biofilm maturation.

From real-time SCFA profiles, the predominance of lactic acid (~63% of total SCFAs) and the reduced formation of secondary fermentation products indicate a delayed metabolic transition from early aerobic metabolism to anaerobic cross-feeding. This interpretation is supported by the post-formation SCFA profiles, which revealed significantly lower levels of formic and lactic acid on LN-functionalized Y-TZP compared with unmodified controls. Together with the lower abundance of *Veillonella*, these findings demonstrate that laminin coatings prolong the reversible adhesion phase and limit the establishment of anaerobic microniches that drive biofilm maturation.

By contrast, FN-coated and unmodified specimens displayed microbial communities and SCFA profiles consistent with unaltered biofilm succession. FN coatings primarily serve as cellular conditioners, enhancing fibroblast adhesion, and matrix deposition via α5β1 integrins [29,50], but exert limited influence on bacterial community structure or metabolism. LN coatings, however, appear to act at both the cellular and microbial level: by promoting fibroblast adhesion while simultaneously restricting metabolic cooperation among bacteria.

The reduced production of butyric acid—commonly associated with late anaerobic taxa such as *Porphyromonas*, *Prevotella,* and *Fusobacterium* [63]—further supports the conclusion that laminin-functionalized surfaces delay ecological progression toward a mature, dysbiotic biofilm. Collectively, these results demonstrate that LN-based ECM coatings do not merely reduce bacterial adhesion but transiently reshape early microbial community structure and metabolic activity toward a host-compatible phenotype.

### Comparison with other surface modification strategies

The biofilm-modulating effects of ECM-based coatings should be considered in the context of existing surface modification strategies for dental implants. Conventional antimicrobial approaches, including metal- or oxide-based coatings (e.g. Ag, Zn, Cu, TiO₂–Ag composites), can effectively suppress bacterial growth but are frequently associated with ion release, cytotoxicity, and limited long-term stability under physiological conditions [64–66]. Physical and topographic surface modifications—such as laser texturing, anodization or plasma treatment—have been shown to improve epithelial and fibroblast attachment, yet primarily act as passive modulators of adhesion rather than biologically selective interfaces [5,6].

In contrast, ECM-derived coatings, particularly crosslinked laminin, represent a biologically guided strategy that mirrors natural tissue interfaces. Rather than exerting bactericidal effects, these coatings promote host-cell adhesion via receptor-specific motifs while transiently disfavoring early microbial colonization.

This concept shifts the focus from antimicrobial suppression toward microbiome modulation, aligning surface design with ecological principles of host–microbe interaction. By supporting a host-preferential interface while delaying early biofilm maturation, ECM-based coatings offer a dual-function strategy that integrates soft-tissue compatibility with biologically selective control of early biofilm development.

### Limitations and future directions

Several limitations should be acknowledged. The static in vitro model does not capture salivary flow, immune responses or mechanical loading, all of which influence biofilm development in vivo. Moreover, the four-day observation period was restricted to early biofilm formation and does not address long-term maturation, coating degradation or sustained microbial adaptation. Accordingly, the present findings should be interpreted as evidence of early-phase modulation rather than persistent biofilm suppression.

Given that late implant loss results primarily from chronic peri-implant inflammation, further research is required to determine whether this early-phase effect influences long-term peri-implant stability. Furthermore, only one laminin variant (cat. no. L4544) and one fibronectin (cat. no. 341635) variant were analyzed. Isoform-specific effects, coating density, and crosslinking chemistry may differentially affect host–microbe interactions and warrant systematic comparison, with respect to connective-tissue integration and epithelial sealing. Material-dependent effects also merit further investigation, as zirconia appeared to stabilize ECM coatings more effectively than titanium under the present conditions.

Ultimately, in vivo validation will be essential to evaluate long-term biostability, host-tissue integration, and microbiome adaptation. Combining ECM-based coatings with complementary immunomodulatory or peptide-based strategies may further enhance peri-implant resilience and translational relevance.

It is worth mentioning that implant abutments are exposed to harsh sterilization procedures (e.g. gamma irradiation or ethylene oxide treatment), as well as mechanical loading, cleaning, and intraoral abrasion during daily use. Previous studies have shown that such sterilization procedures can affect the structural and biochemical properties of biomaterial surfaces, depending on material composition and treatment conditions [67–69]. However, bioinspired coatings based on silane-mediated functionalization have also been reported to exhibit great stability properties under various conditions [30]. Thus, the present findings imply successful laminin functionalization under controlled conditions; the long-term stability of the bioactive layer under clinical sterilization still needs to be investigated. Studies addressing post-sterilization and remaining protein integrity will be essential to further strengthen the translational relevance of this approach.

## Conclusion

This study demonstrates that covalently immobilized extracellular matrix (ECM) proteins on implant abutment materials can modulate early oral biofilm formation. Among all tested configurations, laminin-functionalized yttria-stabilized zirconia most effectively delayed early oral biofilm maturation, reduced metabolic cooperation and promoted a host-favorable microbial profile.

These effects were not bactericidal but reflected transient biological steering of early biofilm formation, highlighting that ECM proteins can selectively modulate host–microbe interactions during the critical initial colonization phase. While the observed effects were confined to early biofilm development in this in vitro model, they underscore the potential of ECM-based coatings as biointerfaces that mitigate early biofilm establishment.

Such biologically selective, microbiome-conscious surface designs may represent a promising strategy to improve peri-implant health and warrant further investigation under dynamic and in vivo conditions to assess long-term stability and clinical relevance.

## Figures and Tables

**Figure A1.**
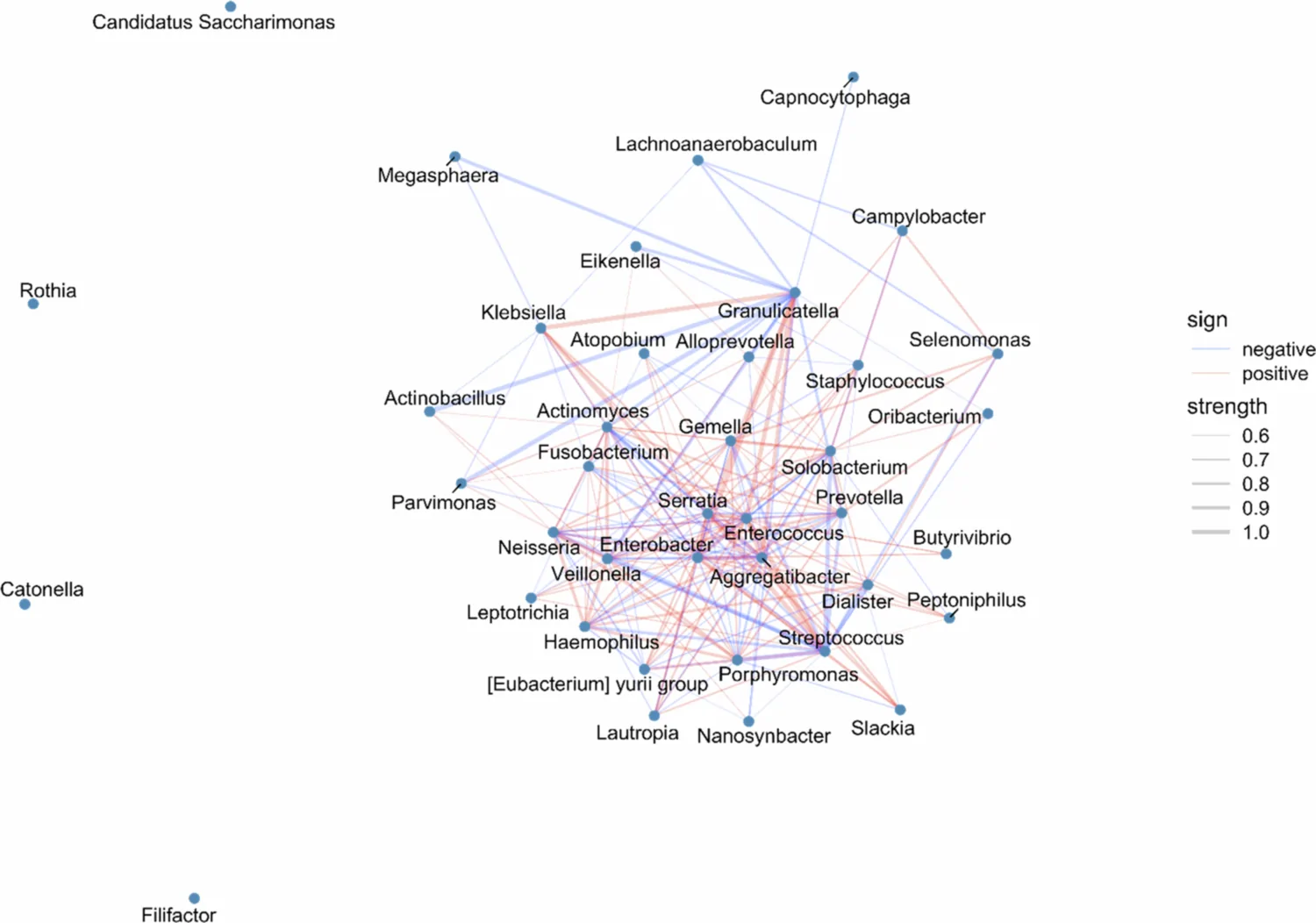
Bacterial association networks of biofilm on Y-TZP constructed using SparCC correlation coefficients for compositional data retaining ASVs with at least 0.1%. A correlation threshold of 0.5 was applied in visualization. Positive correlations were visualized as red lines and negative correlations as blue lines.

**Figure A2.**
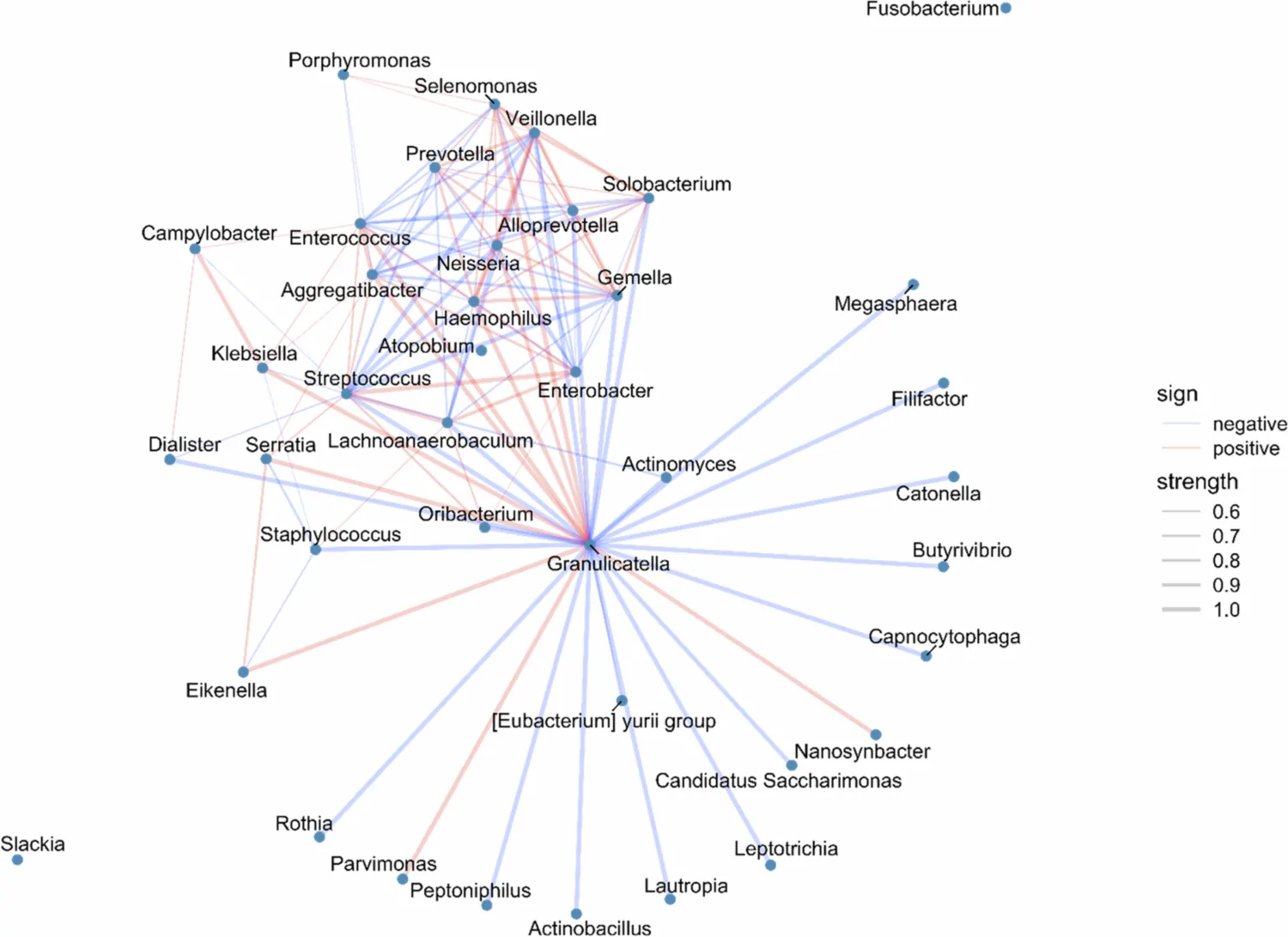
*Bacterial association networks of biofilm on Y-TZP-FN constructed using SparCC correlation coefficients for compositional data, retaining ASVs with abundance at least 0.1%.* A correlation threshold of 0.5 was applied in visualization. Positive correlations were visualized as red liness and negative correlations as blue lines.
